# Pharmacological target therapy of neuropathic pain and patient-reported outcomes in patients with chronic low back pain in Korea: Results from the NLBP Outcomes Research

**DOI:** 10.1097/MD.0000000000011919

**Published:** 2018-08-21

**Authors:** Jae Taek Hong, Jin-Hwan Kim, Keun-Su Kim, Chong-Suh Lee, Hyun-Chul Shin, Woo-Kyung Kim, Joo-Han Kim, Jung-Kil Lee, In-Soo Kim, Yoon Ha, Soo-Bin Im, Sang Woo Kim, In-Ho Han, Jun-Jae Shin, ByeongCheol Rim, Kyung-Soo Suk, Jin-Hyok Kim, Ye-Soo Park, Bong-Soon Chang, Deuk Soo Jun, Young-Hoon Kim, Jung-Hee Lee, Woo-Kie Min, Jung Sub Lee, Si-Young Park, In-Soo Oh, Jae-Young Hong, Bo-Jeong Seo, Young-Joo Kim, Juneyoung Lee

**Affiliations:** a Department of Neurosurgery, The catholic university of Korea, St. Vincent's hospital & Eunpyung St. Mary's Hospital, Suwon; b Department of Orthopedic Surgery, Inje University Ilsan Paik Hospital, Gyeonggi-do; c Department of Neurosurgery, Gangnam Severance Hospital, Yonsei University Health System; d Department of Orthopedic Surgery, Samsung Medical Center, Sungkyunkwan University School of Medicine; e Department of Neurosurgery, Kangbuk Samsung Hospital, Seoul; f Department of Neurosurgery, Gachon University Gil Medical Center, Incheon; g Department of Neurosurgery, Korea University Guro Hospital, Seoul; h Department of Neurosurgery, Chonnam National University Hospital, Kwangju; i Department of Neurosurgery, Keimyung University Dongsan Hospital, Daegu; j Department of Neurosurgery, Severance Hospital, Yonsei University Health System, Seoul; k Department of Neurosurgery, Soonchunhyang University Hospital Bucheon, Gyeonggi-do; l Department of Neurosurgery, Yeungnam university Hospital, Daegu; m Department of Neurosurgery, Pusan National University Hospital, Busan; n Department of Neurosurgery, Inje University Industry Academic Cooperation Foundation, Wonju, Korea; o Department of Neurosurgery, Sun Medical Center, Kerala, India; p Department of Orthopedic Surgery, Gangnam Severance Hospital, Yonsei University Health System; q Department of Orthopedic Surgery, Inje University Sanggye Paik Hospital, Seoul; r Department of Orthopedic Surgery, Guri Hospital, Hanyang University College of Medicine, Gyeonggi-do; s Department of Orthopedic Surgery, Seoul National University Hospital, Seoul; t Department of Orthopedic Surgery, Gachon University Gil Medical Center, Incheon; u Department of Orthopedic Surgery, Seoul St. Mary's Hospital of the Catholic University of Korea; v Department of Orthopedic Surgery, Kyung Hee University Hospital, Seoul; w Department of Orthopedic Surgery, Kyungpook National University Hospital, Daegu; x Department of Orthopedic Surgery, Pusan National University Hospital, Busan; y Department of Orthopedic Surgery, Korea University Anam Hospital, Seoul; z Department of Orthopedic Surgery, Incheon St. Mary's Hospital of the Catholic University of Korea, Incheon; aa Department of Orthopedic Surgery, Korea University Ansan Hospital, Gyeonggi-do; bb Outcomes Research/Real World Data, Corporate Affairs & Health and Value, Pfizer Pharmaceuticals Korea Ltd.; cc Department of Biostatistics, College of Medicine, Korea University, Seoul, Republic of Korea.

**Keywords:** chronic low back pain, neuropathic pain, pharmacological targeted therapy, quality of life

## Abstract

A number of studies have demonstrated an association of neuropathic pain and chronic low back pain (CLBP), but the outcome difference in each medical management is poorly understood. This study is aimed to investigate treatment patterns of neuropathic pain in CLBP patients and to explore patient-reported outcomes (PROs) including quality of life (QoL) and functional disability by treatment patterns.

Data were extracted from the neuropathic low back pain (NLBP) outcomes research. It was a multicenter and cross-sectional study in which 1200 patients were enrolled at 27 general hospitals, from 2014 to 2015. Of total, 478 patients classified as neuropathic pain were used for this subgroup analysis. The patients were divided into 2 groups according to treatment patterns (with vs. without the targeted therapy [TT] of neuropathic pain). Demographic and clinical features were collected by chart reviews and PROs were measured by patient's survey. QoL was assessed by EuroQoL 5-dimension (EQ-5D) questionnaire. Functional disability was measured by the Quebec Back Pain Disability Scale (QBPDS). Multiple linear regression analyses were conducted to compare the PROs between TT group and non-targeted therapy (nTT) group.

Among the NLBP patients (mean age 63years, female 62%), EQ-5D index, EuroQoL-Visual Analog Scale (EQ-VAS), and QBPDS Scores (mean ± standard deviation) were 0.40 ± 0.28, 54.98 ± 19.98, and 46.03 ± 21.24, respectively. Only 142 (29.7%) patients had pharmacological TT of neuropathic pain. Univariate analyses revealed no significant mean differences between TT group and nTT group in the EQ-5D index (0.41 ± 0.27 and 0.39 ± 0.28), EQ-VAS (56.43 ± 18.17 and 54.37 ± 20.69), and QBPDS (45.31 ± 21.32 and 46.31 ± 21.24). After adjustment with covariates, TT group had higher scores of EQ-5D index (β = 0.07; *P* < 0.01) and EQ-VAS (β = 4.59; *P* < 0.05) than the nTT group. The TT group's QBPDS score was lower than the nTT group, although its statistical significance still has not been reached (β = −4.13; *P* = 0.07).

We found that considerable proportion of the NLBP patients remains untreated or undertreated. Although TT group had significantly better QoL than nTT group, only 29.7% of NLBP patients had pharmacological TT. Therefore, clinicians should consider using TT for better QoL of neuropathic pain patients.

## 1 Introduction

Low back pain (LBP) is one of the most common musculoskeletal disorders, resulting in significant personal, social, and economic burden. Mechanical conditions of the spine, including disk disease, disk herniation, spondylosis, spinal stenosis, and fractures, account for up to 98% of LBP cases. Neuropathic pain in chronic LBP was reported to be highly prevalent and neuropathic pain affects the social and psychological well-being of LBP patients.^[1–20]^ A recent systematic review to evaluate prevalence rate of the neuropathic pain in LBP patients has reported prevalence ranging from 29.4 to 73%.^[1–20]^ In addition, the meta-analysis of 20 studies, including a total of 14,269 patients with LBP, found that the pooled prevalence rate of neuropathic low back pain (NLBP) was 47% (40%–54%).^[21]^ Thus, NLBP may require to be considered as an important clinical problem.

Neuropathic pain profoundly decreased the quality of life (QoL).^[22]^ Hiyama et al^[18]^ reported that NLBP patients had significantly higher visual analog scale (VAS) scores and lower the scores of short form 36 (SF-36) and Japanese Orthopedic Association Back Pain Evaluation Questionnaire than LBP patients with nociceptive pain. This result suggests that NLBP affects the physical, social, and psychological well-being compared to nociceptive LBP patients. Hence, it is significant to identify the involvement of neuropathic pain in LBP patients and to effectively manage NLBP.

However, there was no multicenter cohort study not only for the prevalence of NLBP patients but also for the treatment pattern in Korean NLBP patients. Although treatment pattern and outcome could be different in each different country and health care system, it would be valuable to understand relationship between the pattern and the outcome of NLBP treatment.

Pharmacotherapy is the primary clinical approach for managing NLBP. Canadian pain society provided guidelines of pharmacological management of chronic neuropathic pain as follows: first-line treatments were specific antidepressants (tricyclics) and anticonvulsants (gabapentin and pregabalin).^[23]^ Serotonin noradrenaline reuptake inhibitors and topical lidocaine were recommended as second-line therapies. Third-line therapies included tramadol and controlled release opioid analgesics. Recommended forth-line treatments were cannabinoids, methadone, and anticonvulsants. The special interest group of the international association for the study of neuropathic pain recently suggested guidelines of pharmacological management of neuropathic pain.^[24]^ According to their guidelines, tricyclic antidepressants (TCAs), gabapentin, pregabalin, and topical lidocaine were recommended as first-line treatment options and second-line treatments included opioid analgesics and tramadol.

Although these guidelines for neuropathic pain management were provided, there was still a lack of available data on treatment patterns of NLBP patients and outcome by treatment pattern.

Therefore, the purpose of this study was to investigate the treatment patterns of NLBP in Korea and to explore the patient-reported outcomes (PROs) including QoL and functional disability by the treatment patterns.

## 2 Materials and methods

### 2.1 Study design and population

This was a subgroup analysis of chronic low back pain (CLBP) patients with neuropathic pain derived from the NLBP outcome research (OR) that was multicentered, cross-sectional study. Data were collected between December 2014 and May 2015 from 27 nationwide general hospitals of South Korea. This study was approved by the all participated centers’ Institutional Review Board. We included CLBP patients who have moderate degree of LBP at least (VAS > 4) and received “minimally adequate treatment” with a medication trial lasting at least 4 weeks. Patients judged by physicians to meet the following criteria were included: age 20 years; CLBP at least 3 months; patients diagnosed with LBP owing to herniated disc, stenosis, spondylosis, spondylolysis, spondylolisthesis, or degenerative disc disease, according to magnetic resonance imaging or computed tomography findings; VAS at least 4; pain medication at least 4 weeks before the enrollment; and patients who were able to understand and willing to complete the subject information sheet and informed consent form. If the patients had following criteria, they were excluded: cancer, sprain, infection, fracture, ankylosing spondylitis, myofascial pain, or sacroiliitis; surgery within 3 months; current participation in other interventional studies; or patients with a critical or unstable health condition. The patients were clearly informed about the aim of our study, and their informed consents were obtained.

**Figure 1:**
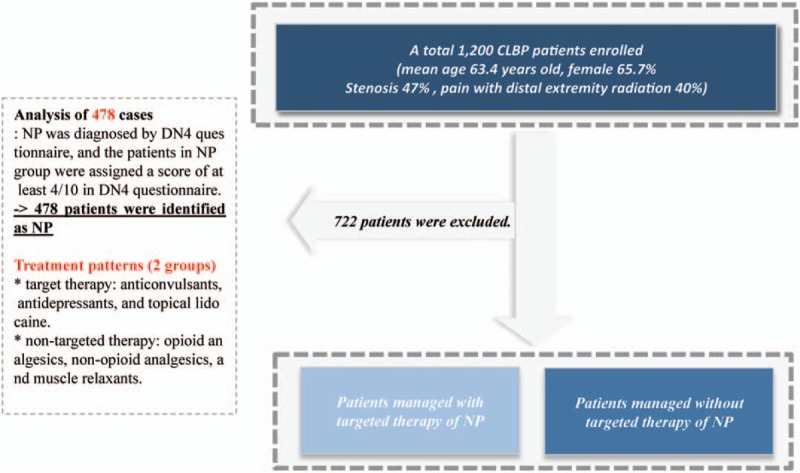
Study design of this study. Among the total 1200 patients enrolled in neuropathic low back pain outcome research, neuropathic low back pain patients whose scores were at least 4/10 in DN4 questionnaire were included in this subgroup analysis. CLBP = chronic low back pain, NP = neuropathic pain.

A target sample size was estimated based on the assumption that the prevalence of NLBP is 37%.^[3]^ With a significance level of 0.05 and an estimated error rate of 2.8%, the required number of patients to be enrolled was calculated to be approximately 1200: 
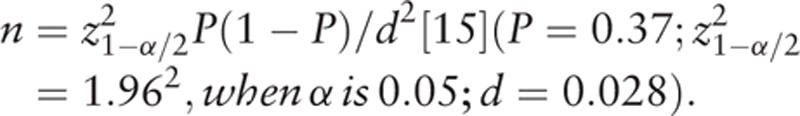


Among the total 1200 patients enrolled in NLBP OR, NLBP patients whose scores were at least 4/10 in Douleur Neuropathique 4 (DN4) questionnaire were included in this subgroup analysis (Fig. 1).

### 2.2 Study data

#### 2.2.1 Baseline variables

Demographic and clinical features were obtained through reviews of medical records and PRO (QoL and functional disability) were measured by the patient's survey. Age, sex, height, and body weight were included in demographic information. Clinical variables contained diagnosis of LBP, comorbidities, symptom period, pain VAS scores, DN4 score, Quebec Task Force Classification for Spinal Disorders (QTFC-SD), and pain control state (pharmacotherapy and surgery). As one of clinical characteristics, QTFC-SD was used to categorize patients’ spinal disorders on the basis of clinical examination and pain localization.^[25]^

#### 2.2.2 Treatment group

Treatment patterns were divided into 2 groups according to whether patients received targeted therapy (TT) of neuropathic pain or not. The TT group included anticonvulsants, TCAs, and topical lidocaine, whereas non-targeted therapy (nTT) group included opioid analgesics, nonopioid analgesics, and muscle relaxants.^[26,27]^

#### 2.2.3 Outcomes

QoL was assessed by generic EuroQoL 5-dimension (EQ-5D) questionnaire. EQ-5D is made up of 2 parts: a descriptive system, which could be converted into a single index, and a EuroQoL Visual Analogue Scale (EQ-VAS). The descriptive measurement consists of 5 dimensions: “mobility,” “self-care,” “usual activities,” “pain/discomfort,” and “anxiety/depression.” Each dimension has 3 levels: no problems, some problems, and severe problems, coded as numbers of 1, 2, and 3 in order. These scores, measured by descriptive part, were converted into scores of a single index (EQ-5D index) with a range from −0.229 to 1 point by applying the equation as follows:

Final EQ-5D index's score^[28]^ = 1 – (0.165 + 0.003 × M2 + 0.274 × M3 + 0.058 × SC2 + 0.078 × SC3 + 0.045 × UA2 + 0.133 × UA3 + 0.048 × PD2 + 0.130 × PD3 + 0.043 × AD2 + 0.103 × AD3 + 0.347 × N3 + 0.014 × I2sq)

The EQ-VAS with a range from 0 to 100 points was used for subjective evaluation of patient's current health state. The ends of the scale were marked “best imaginable health state” and “worst imaginable health.” In these 2 measurements, a higher score means a higher QoL.

Quebec Back Pain Disability Scale (QBPDS) was used for measuring functional disability. The QBPDS consists of 20 questions and has a scale of 0 to 5 for each question with a range from 0 to 100 points. A higher score of this tool indicates a more severe disability of function.

### 2.3 Study ethics

This investigation was designed as multicenter cross-sectional observational study of LBP patients. The patients (or their legal representatives) were provided with all study-related information, and they signed an informed consent form. All participating medical institutions obtained approval from their respective institutional review boards.

### 2.4 Statistical analysis

Patient's demographic and clinical characteristics were summarized as mean ± standard deviation (SD) for continuous variables or frequency (percentage) for categorical variables. Comparisons of patient's characteristics between the TT group and nTT group were made by Student 2 independent sample t test or *χ*^2^ test, as appropriate. Mean differences of patient's QoL measured by EQ-5D index and EQ-VAS scores as well as their functional disability measured by QBPDS were also examined using the independent *t* test. An appropriateness of a use of the test was examined by histogram and normal probability plot for each of numerical variables. For variables with positively skewed data, a logarithmic transformation was performed before the *t* test. Multiple linear regression analyses were performed to compare scores of EQ-5D index, EQ-VAS, and QBPDS between 2 groups after adjusting potential confounders. Variables with *P* < .1 from bivariate analyses were selected as the potential confounders. Degree of performance of the regression model was measured by its coefficient of determination (*R*^2^). Collinearity among explanatory variables has also been checked, and no noticeable problem found in the multiple regression models used in this study. All statistical analyses were performed using the SAS software, version 9.4 (SAS Institute Inc., Cary, NC), and a 2-tailed *P* value <.05 was considered as statistically significant.

## 3 Results

### 3.1 Characteristics of the NLBP patients

Demographic and clinical features of NLBP patients are presented in Table 1. Of a total 478 patients with NLBP, 294 (61.5%) patients were females and mean age was 62.96 ± 13.40 years. The most common type of spinal disease was stenosis (65.7%), followed by herniated disc (38.3%) and spondylolisthesis (15.5%). By the QTFC-SD category, the pain with distal extremity radiation was the most (49.8%). Only 142 (29.7%) patients had pharmacological TT of neuropathic pain. There were not significantly difference between the TT group and the nTT group in terms of age, sex ratio, comorbid diseases, pain VAS, and pain duration. LBP period of TT group (18.30 ± 22.62 months) was significantly longer than the nTT group (14.03 ± 23.99 months). The QTFC-SD items showed significant difference among the groups (*P* < .0001). In the TT group, the most frequent pain type was pain with distal extremity radiation (49.3%) followed by spinal stenosis (16.2%) and spinal nerve root compression (14.8%). pain with distal extremity radiation (50.0%) showed the highest proportion in the nTT group as in the TT group. However, unlike the TT group, pain with proximal extremity radiation (17.6%) was the secondly highest, followed by pain with radiation and neurologic finding (11.3%).

**Table 1:**
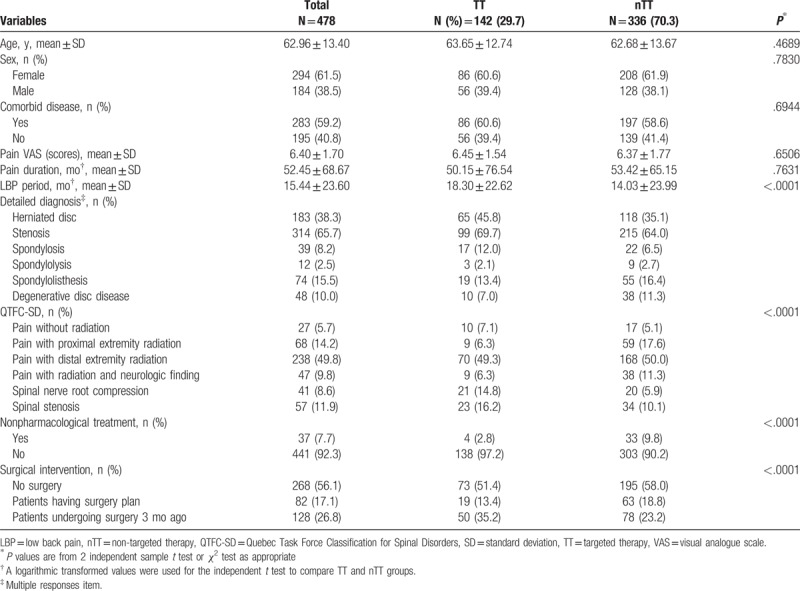
Demographic and clinical variables of patients with neuropathic chronic low back pain (n = 478).

Our data also showed that the subject's severity of LBP measured by QBPDS was associated with their anxiety/depression which was the fifth dimension of EQ-5D index measured as “none,” “some/moderate,” and “extreme.” Specifically, patient having more severe anxiety/depression showed significantly higher mean QBPDS score (*P* < .0001).

### 3.2 PRO in the NLBP patients

**Figure 2:**
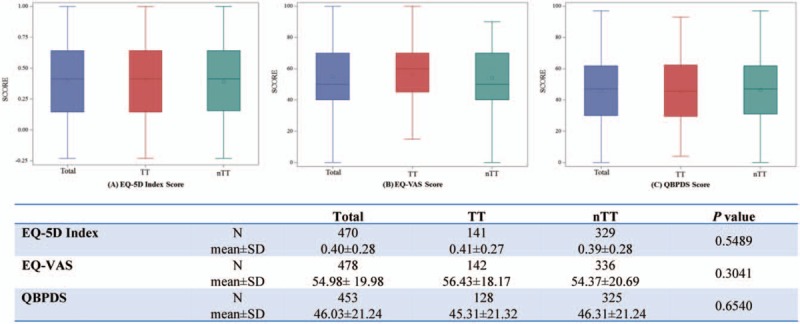
Patient-reported outcome scores in patients with neuropathic chronic low back pain. There were no differences in the EQ-5D index, EQ-VAS and QBPDS between two groups (TT and nTT) in the student t-tests. However, the multiple linear regression analysis showed that EQ-5D index scores and EQ-VAS scores were significantly higher in the patients managed by the TT (*β* = 0.07; *P* < 0.01,*β* = 4.59; *P* < 0.05 respectively) than the nTT group. The patients who received TT tended to have lower QBPDS scores (*β* = −4.13; *P* = 0.07), compared to the nTT group. EQ-5D = EuroQoL 5-dimension, EQ-VAS = EuroQoL Visual Analogue Scale, nTT = non-targeted therapy, QBPDS = Quebec Back Pain Disability Scale, SD = standard deviation, TT = targeted therapy, VAS = visual analogue scale.

In NLBP patients, mean scores of EQ-5D index, EQ-VAS, and QBPDS were 0.40 ± 0.28, 54.98 ± 19.98, and 46.03 ± 21.24, respectively (Fig. 2). In the student *t* tests, performed to identify differences of PRO among 2 groups (TT and nTT), there were no differences in the EQ-5D index (0.41 ± 0.27 and 0.39 ± 0.28), EQ-VAS (56.43 ± 18.17 and 54.37 ± 20.69), and QBPDS (45.31 ± 21.32 and 46.31 ± 21.24) (Fig. 2).

In the multiple linear regression analyses adjusting with potential confounders (age, sex, duration of LBP, QTFC-SD category, comorbidities, scores of DN4, and scores of pain VAS), the patients managed by the TT showed higher scores of EQ-5D index (*β* = 0.07; *P* < .01) (Table 2) and EQ-VAS scores (*β* = 4.59; *P* < .05) (Table 3) than the nTT group. The patients who received TT tended to have lower QBPDS scores (*β* = −4.13; *P* = .07) (Table 4), compared to the nTT group.

**Table 2:**
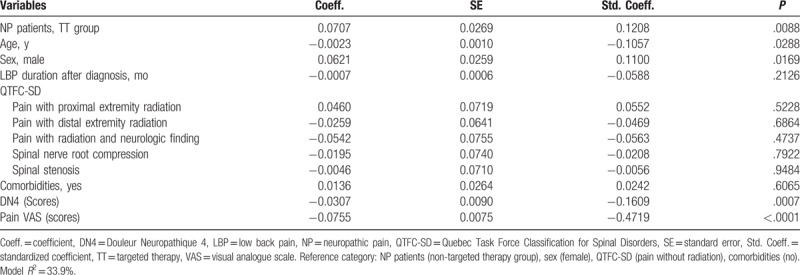
Effect of targeted therapy on patient's quality of life measured by EuroQol-5dimensions index score after adjusting potential confounders with multiple linear regression analysis.

**Table 3:**
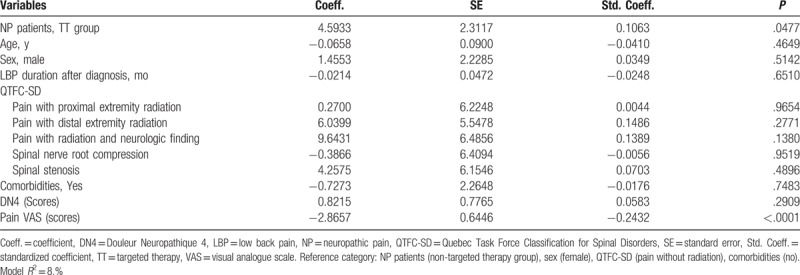
Effect of targeted therapy on patient's quality of life measured by EuroQol-visual analogue scale after adjusting potential confounders with multiple linear regression analysis.

**Table 4:**
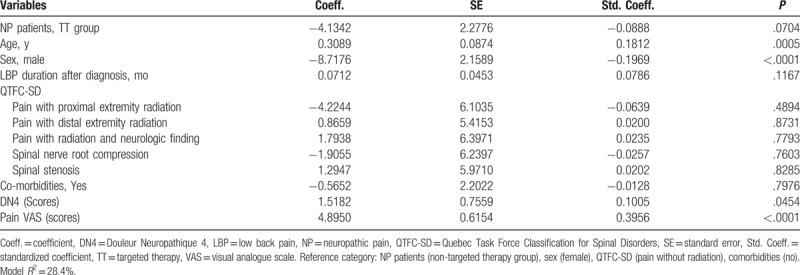
Effect of targeted therapy on patient's functional disability measured by Quebec Back Pain Disability Scale score after adjusting potential confounders with multiple linear regression analysis.

## 4 Discussion

This study showed the mismatch between diagnosis and treatment pattern of NLBP patients. Although 39.8% of LBP patients met the DN4 criteria for neuropathic pain, only 29.7% were receiving pharmacological treatment with proven efficacy in neuropathic pain. The literature reveals that *neuropathic pain* is often *undertreated* or treated with ineffective or untested modalities.

A reason for the low proportion of patients having TT would be difficulty in distinguishing the certain clinical difference between NLBP and nociceptive LBP. Identifying the underlying mechanism of chronic pain allows the use of pharmacological agents targeting specific pain mechanisms.^[29]^ NLBP results from a primary lesion or a malfunction within the somatosensory system, whereas nociceptive LBP is caused by tissue injury and/or inflammatory process.^[30]^ The general clinical diagnosis of neuropathic pain was based on the evidence of a lesion or a disease of the nervous system, which was ascertained by interviewing the patients and performing clinical examinations. However, it is complex and difficult methods to detect neuropathic pain in LBP patients.

Some clinicians used screening tools of neuropathic pain as a simpler means. However, there were various standardized screening measurements incurring wide variation in diagnosis of neuropathic pain and no consensus on the diagnosis of neuropathic pain.

In addition, although there were many evidence-based guidelines in the pharmacological management of neuropathic pain due to the attempts for developing a therapeutic approach of related societies, neuropathic pain treatment guideline for LBP was absent.

Canadian pain society provided guidelines of pharmacological management of chronic neuropathic pain as follows: first-line treatments were specific antidepressants (tricyclics) and anticonvulsants (gabapentin and pregabalin).^[23]^ Serotonin noradrenaline reuptake inhibitors and topical lidocaine were recommended as second-line therapies. Third-line therapies included tramadol and controlled release opioid analgesics. Recommended forth-line treatments were cannabinoids, methadone, and anticonvulsants. The special interest group of the international association for the study of neuropathic pain recently suggested guidelines of pharmacological management of neuropathic pain.^[24]^ According to their guidelines, TCAs, gabapentin, pregabalin, and topical lidocaine were recommended as first-line treatment options and second-line treatments included opioid analgesics and tramadol.

We defined the TT and nTT based on these 2 references. Target therapy included TCAs, gabapentin, pregabalin, and topical lidocaine, which are recommended as first- or second-line medical treatment because their efficacy in neuropathic pain has been established in multiple randomized controlled trials (RCTs).

Neuropathic pain patients had severe and chronic symptoms that impaired their QoL.^[31,32]^ QoL measured by EQ-5D scores of patients with neuropathic pain (0.40 ± 0.28) was considerably lower in this study than those of other chronic diseases reported in the previous study (hypertension: 0.81,^[33]^ 0.87–0.89;^[34]^ cardiovascular disease: 0.74,^[33]^ 0.62–0.72;^[35]^ diabetes: 0.82,^[33]^ 0.83;^[36]^ cancer: 0.86;^[36]^ chronic kidney disease: 0.885;^[37]^ rheumatoid arthritis: 0.83,^[36]^ 0.67–0.73^[38]^), which showed the negative impact of the neuropathic pain in CLBP patients. After adjustment with covariates, the patients managed by pharmacological TT showed higher EQ-5D index and EQ-VAS scores than the nTT group, which suggested that the neuropathic pain patients who received the TT showed significantly better outcome in terms of pain control and QoL than the nTT group. In many RCTs, medications included in TT group of our study were proven in terms of efficacy of neuropathic pain therapy. Two studies of antidepressants in neuropathic pain reported that TCAs provided the identical efficacy in the neuropathic pain management.^[39,40]^ Gabapentin and pregabalin have shown the efficacy in the studies on comparison between anticonvulsants and placebo in patients with several neuropathic pain conditions.^[41,42]^ The 5% lidocaine patch has shown excellent efficacy and tolerability in allodynia patients due to various types of peripheral neuropathic pain.^[41,42]^

This study has several limitations. First, some of the well-known risk factors for NLBP include advanced age, female sex, and diabetes with or without hypertension, obesity, smoking, and psychological factors such as depression. Although these data demonstrated that neuropathic LBP and depression are significantly correlated with each other, the relationship between neuropathic LBP and other risk factors was not examined in this study because the aim of this study was to investigate treatment patterns of NLBP patients and to explore PRO including QoL and functional disability by treatment patterns.

Second limitation of this study stems from the differential diagnosis of neuropathic pain. This study used DN4 questionnaire to diagnose a neuropathic pain in LBP patients based on a more reliable identification and qualification of a neuropathic pain. Despite the good sensitivity and specificity of the DN4 questionnaire, the question remains whether the distinction between neuropathic and nociceptive symptom profiles truly represents the biological background of pain, or whether it may be an artificial effect. Moreover, these categories of pain overlap to some degree. Although screening tools may give guidance to clinicians by selecting patients that need further diagnostic evaluation and pain management by specialists, they clearly do not replace clinical judgment. In this regard, the evaluation method using the questionnaire that was employed in this study is also a limitation.

In addition, it should be noted that because other pain measures were not part of the study, no comparisons can be made between the DN4 questionnaire and other neuropathic pain screening scales.

The third limitation of this study results from the subject population because it was mostly performed at a tertiary care university hospital. The spectrum of presenting patients obviously differs between primary care clinics and community hospitals. Fourth, this subgroup analysis has the limitation on the sample size, not calculated to investigate this subgroup. Thus, it is difficult to generalize the study results. Lastly, we could use only measured variables in this study as the potential confounders.

Despite these limitations, there have been no other similar studies to compare the treatment outcome between TT and nTT in the NLBP population and this study showed that TT could be associated with a better QoL of NLBP patients. There were, however, relatively low numbers of patients having pharmacological TT of neuropathic pain in this study. These results could suggest that in cases with neuropathic pain, appropriate pharmacological treatments for the neuropathic component should be considered to have better QoL. Further studies are required to explore effects of TT on the improvement of QoL and functional ability in larger population with NLBP.

## Author contributions

**Conceptualization:** Bo-Jeong Seo, Young-Joo Kim.

**Data curation:** Jae Taek Hong, Jin-Hwan Kim, Keun-Su Kim, Chong-Suh Lee, Hyun-Chul Shin, Woo-Kyung Kim, Joo-Han Kim, Jung-Kil Lee, In-Soo Kim, Yoon Ha, Soo-Bin Im, Sang Woo Kim, In-Ho Han, Jun-Jae Shin, ByeongCheol Rim, Kyung-Soo Suk, Jin-Hyok Kim, Ye-Soo Park Park, Bong-Soon Chang, Deuk Soo Jun, Young-Hoon Kim, Jung-Hee Lee, Woo-Kie Min, Jung Sub Lee, Si-Young Park, In-Soo Oh, Jae-Young Hong.

**Formal analysis:** Juneyoung Lee.

**Investigation:** Jae Taek Hong, Hyun-Chul Shin, Woo-Kyung Kim, Joo-Han Kim, Jung-Kil Lee, In-Soo Kim, Yoon Ha, Soo-Bin Im, In-Ho Han, Jun-Jae Shin, ByeongCheol Rim, Kyung-Soo Suk, Jin-Hyok Kim, Ye-Soo Park Park, Bong-Soon Chang, Deuk Soo Jun, Young-Hoon Kim, Jung-Hee Lee, Woo-Kie Min, Jung Sub Lee, Si-Young Park, In-Soo Oh, Jae-Young Hong.

**Methodology:** Jae Taek Hong, Bo-Jeong Seo.

**Project administration:** Bo-Jeong Seo.

**Supervision:** Keun-Su Kim, Chong-Suh Lee, Bo-Jeong Seo, Young-Joo Kim.

**Validation:** Jae Taek Hong, Jin-Hwan Kim, Bo-Jeong Seo.

**Visualization:** Bo-Jeong Seo.

**Writing – original draft:** Jae Taek Hong, Jin-Hwan Kim.

**Writing – review & editing:** Keun-Su Kim, Chong-Suh Lee, Bo-Jeong Seo, Young-Joo Kim, Juneyoung Lee.
